# Adenoid basal carcinoma of the cervix in a 20-year-old female: a case report

**DOI:** 10.1186/1746-1596-1-20

**Published:** 2006-08-16

**Authors:** William David DePond, Victor Santos Flauta, Daniel Christian Lingamfelter, David Mark Schnee, Kristyn Poncy Menendez

**Affiliations:** 1 Department of Pathology, University of Missouri-Kansas City School of Medicine and Truman Medical Centers, Kansas City, Missouri, USA; 2 Department of Obstetrics and Gynecology, University of Missouri-Kansas City School of Medicine and Truman Medical Centers, Kansas City, Missouri, USA

## Abstract

**Background:**

Adenoid basal carcinoma of the cervix is a rare condition mostly occurring among postmenopausal women. Although it can be confused with adenoid cystic carcinoma of the cervix, adenoid basal carcinoma has several clinicopathologic features that will allow distinction from adenoid cystic carcinoma.

**Case presentation:**

This is the case of a twenty-year old African-American female who initially presented with a high-grade squamous intraepithelial lesion on Pap smear, with a subsequent cervical LEEP specimen revealing adenoid basal carcinoma. The lesion showed the characteristic histologic features of adenoid basal carcinoma and was positive for the immunohistochemical marker EMA and negative for collagen IV, further defining the tumor while helping to rule out the possibility of adenoid cystic carcinoma. As far as the authors are aware, this is the youngest reported case of adenoid basal carcinoma to date.

**Conclusion:**

This case shows that adenoid basal carcinoma can deviate markedly from its typical postmenopausal demographics to affect women as young as 20 years of age. In addition, adenoid basal carcinoma has several identifiable features that will differentiate it from adenoid cystic carcinoma including histologic and cellular morphologies, as well as immunohistochemistry. Treatment for most patients involves hysterectomy, LEEP, or a conization procedure which provides a favorable prognosis because of this lesion's low potential for recurrence and metastasis.

## Background

Adenoid basal carcinoma (ABC) of the cervix is a rare neoplasm with a predilection for postmenopausal non-Caucasian women. It accounts for less than 1% of all cervical adenocarcinomas. Since its original recognition in 1966 by Baggish and Woodruff [1,2], there have been fewer than 100 reported cases of this condition (90 as of this writing), with the vast majority occurring among women 45 years of age or older. However, Zamecnik [3] recently reported a case occurring in a 21 year-old female. Herein, we present the occurrence of ABC within the uterine cervix of a 20-year-old female, the youngest reported case of its kind and only the second reported case in a patient younger than thirty years of age.

## Case presentation

A 20-year-old African American female presented with a history of a high-grade squamous intraepithelial lesion (HSIL) on Pap smear. A colposcopy was performed and yielded three cervical biopsies and an endocervical curettage, all of which showed no significant pathologic alteration with exception of one cervical biopsy that showed HPV-effect. Still concerned with the patient's HSIL result, clinicians performed a cervical loop electrosurgical excision procedure (LEEP), during which multiple erosions of inflammation were observed to be overlying the cervix.

### Materials and methods

5-um sections from the formalin-fixed, paraffin-embedded tissue were used for routine light microscopic study as well as immunohistochemical analysis including the antibodies against prostate-specific antigen (PSA) (1:100), epithelial membrane antigen (EMA) (prediluted), and collagen IV (1:40) (DakoCytomation, Glostrup, Denmark). These immunohistochemical stains were performed by a labeled avidin-biotin complex immunoperoxidase method using commercially available monoclonal antibodies and DAB as the chromogen. In order to provide negative controls on patient tissue and thereby ensure specificity of the reactions, the aforementioned antibodies were substituted for an unrelated antibody during the incubation procedure. Formalin-fixed and paraffin-embedded tissue from kidney, prostate, and breast were used as positive controls for collagen IV, PSA, and EMA, respectively.

### Pathologic findings

The gross LEEP specimen measured 1.3 cm in diameter and 0.4 cm in depth. The cervical mucosa was purple-tan, smooth and glistening. No demonstrable lesions were present.

**Figure 1:**
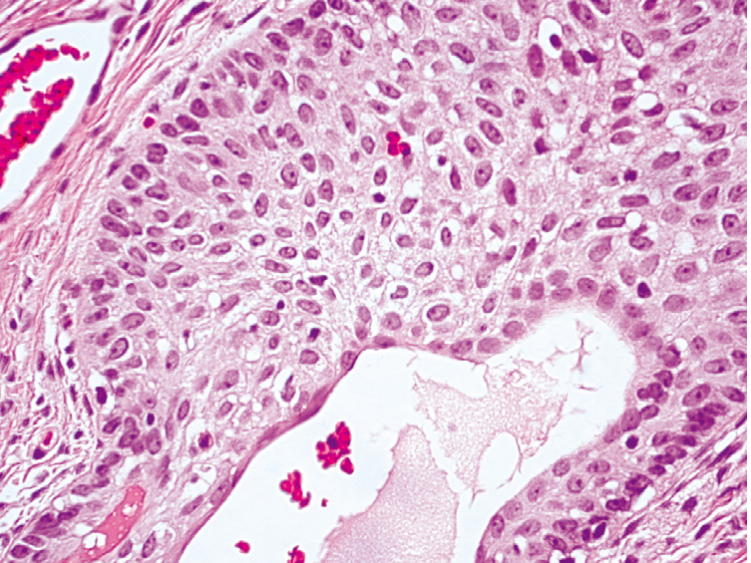
**(H&E, ×100): **A palisading arrangement around the periphery of this cellular nest is striking.

**Figure 2:**
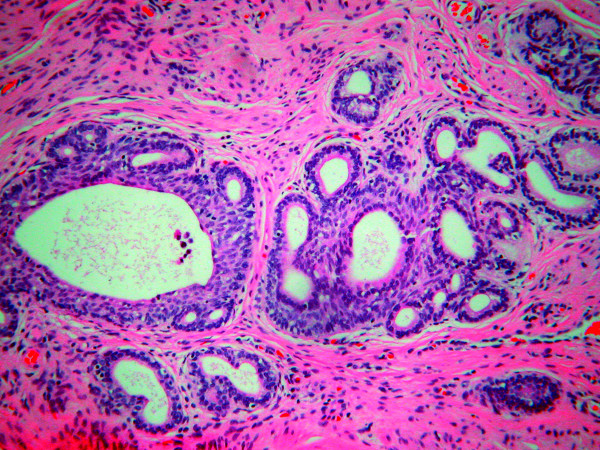
**(H&E, ×10): **The cervical stroma shows scattered cellular proliferations arranged in nests with peripheral palisading and microcyst formation. A stromal reaction to these nests is absent.

Microscopically, hematoxylin- and eosin-stained sections showed cervical stroma involved by scattered cellular proliferations arranged in nests with a striking peripheral palisading arrangement (Fig. 1) and microcyst formation. A stromal reaction to these nests was not present (Fig. 2). The cells were uniform, round to oval, and had scant cytoplasm. Nuclei were hyperchromatic, showing inconspicuous nucleoli and minimal nuclear atypia. Mitotic figures were not identified. The squamous epithelium showed occasional, sporadic foci of lymphocytic infiltration not necessarily coincident to the underlying tumor nests. Definitive squamous intraepithelial lesions were not present.

**Figure 3:**
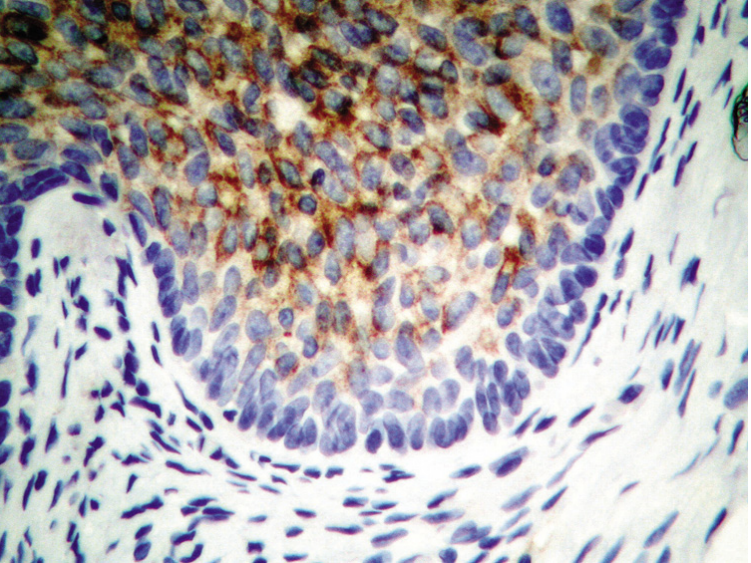
**(EMA, ×40): **Most of the tumor cells within the nests show a strong, diffuse cellular membrane staining. The peripheral palisading cells show a uniform lack of reactivity to the antibody.

In order to further elucidate the nature of the tumor, immunohistochemical stains were performed. A stain for EMA showed strong, diffuse cellular membrane staining of the tumor cells (Fig. 3), whereas stains for PSA and collagen IV did not highlight cells from the lesion. The positive control tissues were diffusely and strongly positive for their respective antibodies.

### Comment

Adenoid basal carcinoma is a rare, indolent lesion of the uterine cervix most commonly occurring in postmenopausal African-American females, although some recent reports have shown that these tumors can also occur in Asian patients [4-6]. Its cellular origin remains uncertain, but immunohistochemical evidence has shown that this lesion may originate from the reserve cell layer of the cervical epithelium [7].

Cviko [8] enumerated the four distinct components of ABC: a classic HSIL; a limited invasive component with squamoid maturation, often with a discrete layer of peripheral basal cells; an outgrowth of small basal cells from either HSIL or squamoid areas; and focal endocervical (adenoid) differentiation.

Histologically, ABC has been described as a proliferation of nested uniform, bland basaloid cells with a peripheral palisading pattern. Mild chromatin abnormalities are present with small nucleoli and rare mitoses. Cytologically, the neoplasm appears as discohesive groups of intact cells with overlapping nuclei, moderately high nuclear-to-cytoplasm ratio, occasional peripheral palisading, finely granular chromatin, mild hyperchromasia, and small nucleoli [9].

Immunohistochemical characteristics include stain positivity for CK14, CK17, CK19 [3] and Cytokeratin KL 1 [9]. Of the nine cases of ABC studied by Grayson [10], all were positive for EMA but totally negative for collagen IV and laminin, compared to adenoid cystic carcinoma (ACC) which was positive for all three stains. Unfortunately, we were unable to perform a laminin stain on the tissue for this case, but the fact that collagen IV alone did not highlight any of the tumor cells (and again taking into consideration the architectural and cytologic features of the tumor) provides solid evidence that this lesion is indeed adenoid basal carcinoma, and not ACC.

Genetic studies have shown correlation with viral infection. Takeshima [6] correlated the occurrence of ABC to human papilloma virus infection. Jones [11] characterized the presence of HPV type 16 among the five cases he studied. Moreover, p53 gene alterations including wild type hyperexpression and p53 point mutational damage were noted. Our patient showed HPV-effect on a cervical biopsy, but the LEEP specimen revealed neither HPV changes nor squamous intraepithelial lesions.

The overwhelming majority of cases are benign owing to the low potential for recurrence and metastasis. This has led to an alternative designation, adenoid basal epithelioma. Brainard [12] and colleagues, in their review of 12 cases, pointed out that adenoid basal carcinoma with typical features is not a malignant neoplasm, does not produce a visible lesion, does not metastasize, and never itself has caused death. Thus, they proposed that the name be changed to reflect the neoplasm's benignity.

Because of its favorable prognosis, this lesion should be distinguished from more malignant processes such as ACC, which carries a recurrent and metastatic potential; adenoma malignum (minimal deviation adenocarcinoma); and mesonephric adenocarcinoma. The most important of these to consider is ACC, which histologically can appear very similar to ABC. Furthermore, they both show positive reactivity to CEA, EMA, CK7, CK20, and CAM 5.2 [10]. However, cellular pleomorphism, mitoses, necrosis, stromal hyalinization, and metastasis are commonly identified in ACC but are rare or absent in ABC. Moreover, as previously discussed, immunohistochemical studies show positive reactivity to collagen IV and laminin for ACC whereas these stains do not highlight the tumor cells of ABC [10].

Other entities to consider within the differential diagnosis include small cell carcinoma of the cervix, carcinoid tumor of the cervix, carcinosarcoma of the cervix, and ectopic prostatic tissue. The last of these was ruled out in our case by using a PSA immunohistochemical marker. Again, histologic, morphologic, and immunohistochemical characteristics of ABC should distinguish it from these other entities.

## Conclusion

This tumor represents the youngest reported occurrence of adenoid basal carcinoma of the cervix. This case, along with that reported by Zamecnik [3], shows that ABC may not always conform to its usual demographics, affecting women as young as their early twenties. Clinicians and pathologists alike are thereby urged to expand their age demographics for this cervical neoplasm.
